# Notes and News

**Published:** 1904-01-16

**Authors:** 


### Notes and News.

The Royal Victoria Hospital, Belfast, has received a gift of £10,000 from the Misses Riddel, in memory of their brother, the late Mr. Samuel Riddel.

In consequence of the great increase in the number of pneumonia cases in New York the Board of Health is considering whether pneumonia should not be added to the list of notifiable diseases.

We learn that the Index Medicus has now 450 subscribers, and if this small number is increased by only 50 the Carnegie Institute in New York will probably place it on a permanent basis.

The late Mr. Forster Green, of Belfast, has bequeathed £10,000 to the Forster Green Hospital for Consumption, subject to his wife's life interest in the sum. Mr. Forster Green founded the hospital in his lifetime.

A new galloping ambulance invented by Mr. Mills, of an American firm, has been tested in the training of a number of battalions of Imperial Yeomanry, and with improvements recently added has proved most satisfactory. The main recommendation is that the car can be utilised more expeditiously and with less oscillation than any other.

A Suffolk man sent two carcases of pigs to London for sale, knowing them to be unfit for food. The carcases were seized, and at the Guildhall Alderman Burnett, before whom the case was brought, sentenced the offender to three weeks' hard labour. The Alderman explained that an impression was current that a fine was imposed in such cases, but that at his Court imprisonment was the rule. In the present instance he remarked that had the offender been a man of better education he should have imposed six months' imprisonment.

The report circulated that Jamaica was suffering from yellow fever is denied by the Governor of the colony.

Through the will of the late Mr. John Penn, M.P. for Lewisham, the Miller Hospital, Greenwich, of which Mr. Penn was treasurer, will receive £2,000.

Battersea has decided to employ a female sanitary inspector, at a salary of £110 per annum, to instruct mothers how to diet children and nurse them in illness.

The annual meeting of the Royal Waterloo Hospital for Children and Women, will be held at 3 p.m. on January 18, at the Mansion House, the Lord Mayor presiding.

The last of the three negroes under treatment for sleeping sickness at the Hospital des Dames Françaises, Paris, is dead. The Academie de Médicine is conducting the scientific examination of the body.

The late Mr. Philip Hedgcock, of Brighton, has bequeathed £1,000 to the Sussex County Hospital, £300 each to the Brighton, Hove, and Preston Dispensary, the Sussex Eye Hospital, and Brighton Homœopathic Hospital. Mr. Hedgcock has left a large sum of money at the discretion of the civic authorities of Brighton for charitable purposes.

Three original plays, specially written by officers of the Channel Fleet, will be produced at the Queen's Gate Hall, South Kensington, on Wednesday next, January 13th, at 8 p.m., on behalf of the Royal Waterloo Hospital for Children and Women. Tickets and all information can be obtained at the hospital from the Secretary, or from A. L. Prynne, Esq., 6 Comeragh Road, West Kensington.

Sir John Craggs, in a letter to the *Times*, protests against the scheme proposed by the Asylums Board, to utilise some of their small-pox hospitals for the reception of tuberculous cases. He points out that these hospitals were erected on the plea that they were a desirable precaution against epidemics and that this precaution would be rendered useless by the scheme. And he argues that the Board having shown such inconsistency of policy and proved so expensive in their methods should not be entrusted with provision for the tuberculous poor of the Metropolis.

Mrs. Garrett Anderson takes advantage of Sir John Cragg's inference that the building of the small-pox hospitals is proved unnecessary because in Berlin an extraneous hospital was converted into a general hospital. She points out that the conditions are not the same because in Berlin there is no probability of a great epidemic as vaccination and re-vaccination is enforced, whereas London is not so protected. Therefore, Mrs. Garrett Anderson points out that the Asylums Board is practically forced to incur the enormous expenditure, in order to be ready for an epidemic possible at any moment.

